# Effects and mechanisms of puerarin against neuroblastoma: insights from bioinformatics and in vitro experiments

**DOI:** 10.1186/s12906-024-04569-0

**Published:** 2024-07-09

**Authors:** Xiaohui Sui, Tingting Liu, Zhiyun Zou, Baoqing Zhang, Guiju Zhang

**Affiliations:** 1 Shandong University of Traditional Chinese Medicine, Jinan Shandong, 250014 China; 2 Affiliated Hospital of Shandong, University of Traditional Chinese Medicine, Jinan Shandong, 250011 China

**Keywords:** Neuroblastoma, Puerarin, Bioinformatics, Gap junction

## Abstract

**Background:**

Neuroblastoma, a prevalent solid tumor in children, often manifests with hidden onset sites, rapid growth, and high metastatic potential. The prognosis for children with high-risk neuroblastoma remains poor, highlighting the urgent need for novel prognostic models and therapeutic avenues. In recent years, puerarin, as a kind of small molecule drug extracted from Chinese medicine Pueraria lobata, has demonstrated significant anticancer effects on various cancer cell types. In this study, through bioinformatics analysis and in vitro experiments, the potential and mechanism of puerarin in the treatment of neuroblastoma were investigated, and a prognostic model was established.

**Methods:**

A total of 9 drug-disease related targets were observed by constructing a database of drug targets and disease genes. Besides, GO and KEGG enrichment analysis was performed to explore the potential mechanism of its therapeutic effect. To construct the prognostic model, risk regression analysis and LASSO analysis were carried out for validation. Finally, the prognostic genes were identified. Parachute test and immunofluorescence staining were performed to verify the potential mechanism of puerarin in neuroblastoma treatment.

**Results:**

Three prognostic genes, i.e., BIRC5, TIMP2 and CASP9, were identified. In vitro studies verified puerarin's impact on BIRC5, TIMP2, and CASP9 expression, inhibiting proliferation in neuroblastoma SH-SY5Y cells. Puerarin disrupts the cytoskeleton, boosts gap junctional communication, curtailing invasion and migration, and induces mitochondrial damage in SH-SY5Y cells.

**Conclusions:**

Based on network pharmacology and bioinformatics analysis, combined with in vitro experimental verification, puerarin was hereby observed to enhance GJIC in neuroblastoma, destroy cytoskeleton and thus inhibit cell invasion and migration, cause mitochondrial damage of tumor cells, and inhibit cell proliferation. Overall, puerarin, as a natural medicinal compound, does hold potential as a novel therapy for neuroblastoma.

**Supplementary Information:**

The online version contains supplementary material available at 10.1186/s12906-024-04569-0.

## Background

Neuroblastoma, a childhood tumor originating in the sympathetic nervous system, is typically diagnosed around 17 months of age [1]. It is also one of the most common solid tumors in childhood, accounting for about 15 percent of pediatric tumor deaths [2]. The clinical manifestations of neuroblastoma vary according to the location of the tumor and the severity of the disease [3]. Neuroblastoma can occur in various parts of the sympathetic nervous system, including the neck, chest and abdomen. Tumor cell proliferation and metastasis pose significant challenges to treating the disease. Current treatment options for children with neuroblastoma include surgery, chemotherapy, radiation therapy, immunotherapy, and hematopoietic stem cell transplantation (AHSCT) [4]. Despite the high survival rates for children with low and medium-risk neuroblastoma [5], outcomes for children with high-risk neuroblastoma remain poor, with 5-year survival rates of less than 50% [6, 7]. Most at-risk children do not respond to first-line treatment or are at high risk of relapse in the first few years after treatment [8]. Therefore, discovering small molecule targeted drugs to treat neuroblastoma, inhibit tumor cell proliferation and metastasis, and extend the survival cycle of children with the disease is of utmost importance.

As society and medicine progress, Chinese herbal medicine has increasingly proven its worth in disease prevention and treatment. In recent years, numerous medicinal plants and herbal compounds have gained significant attention in tumor drug research. Puerarin, for example, has shown promising intervention capabilities in treating nervous system diseases [9]. It is an isoflavone compound extracted from pueraria root, furnished with many pharmacological effects such as anti-inflammation, anti-cancer, anti-oxidation and anti-ischemia reperfusion injury [10–13]. Previous studies have shown the positive anti-cancer effects of puerarin in a variety of cancers, including prostate cancer[14], lung cancer[15], and oral squamous cell carcinoma [16]. In addition, its protective effect on nervous system diseases by activating PI3K/Akt signaling pathway has also been confirmed, which also delays degenerative diseases of the nervous system [17, 18]. However, the role of puerarin in neuroblastoma remains unclear. To this end, the effects and mechanisms of puerarin in the treatment of neuroblastoma were hereby investigated using network pharmacology and bioinformatics methods combined with in vitro experiments.

## Method

### Potential target acquisition in neuroblastoma

Therapeutically Applicable Research to Generate Effective Treatments (TARGET) (https://ocg.cancer.gov/programs/target) is an open database for childhood cancers. Herein, the RNA sequencing data and corresponding clinical information of 244 neuroblastoma samples were obtained from the TARGET database, of which 213 were in the COG high risk group and 31 were in the COG low risk group. Differentially expressed mRNAs between the COG high and low risk groups were delved into using the limma package in R software[19]. Adjusted *P* < 0.05 and Log1.3 (Fold Change) > 1 or Log1.3 (Fold Change) <  − 1″ were defined as the threshold for the differential expression of mRNAs.

### Drug-disease related targets acquisition

Puerarin was analyzed using the TCMSP database (https://old.tcmsp-e.com/tcmsp.php) to identify known drug targets, and Uniport database was employed to transform and standardize puerarin drug target data to construct a drug target gene database. Besides, Venny 2.1.0 was utilized to map the targets of puerarin alongside known therapeutic targets of neuroblastoma, establishing a Venn diagram for comparative analysis. After removing the repeat target for NB, the puerarin target was intersected with a potential therapeutic target for NB. These cross-over targets are defined as drug-disease related targets for the treatment of NB. Additionally, drug disease composite target network was constructed using Cytoscape (version 3.7.2, Boston, MA, USA). To explore the connections between key targets, the protein–protein interaction network was further mapped using the string database (https:// string-db.org /).

### Functional enrichment

To explore the functions of drug-disease related targets, a functional enrichment analysis was conducted utilizing Gene Ontology (GO). This tool is commonly used to annotate genes with their molecular function (MF), biological pathways (BP), and cellular components (CC). Additionally, Kyoto Encyclopedia of Genes and Genomes (KEGG) Enrichment Analysis was employed as a practical resource for studying gene functions and associated high-level genome functional information. Subsequently, to visualize the results, the ClusterProfiler package (version: 3.18.0) in R was utilized [19].

### Correlation test between genes and survival

Herein, by using gene expression and clinical data from 249 patients extracted from the TARGET database, an investigation was conducted to determine whether any drug-related target genes were linked to survival in neuroblastoma patients. Based on the expression of drug-related target genes, these patients were divided into high and low groups, and 9 genes were individually analyzed. Besides, the log-rank test was conducted to compare survival differences between these groups. Additionally, timeROC (v 0.4) analysis was employed to compare the predictive accuracy of each gene. Subsequently, *p*-values and hazard ratios (HR) with 95% confidence intervals (CI) for Kaplan–Meier curves were generated using log-rank tests and univariate Cox proportional hazards regression. A *p*-value less than 0.05 was considered statistically significant.

### Construction of prognostic model

Based on the RNA sequencing data (level 3) and corresponding clinical information of 249 neuroblastoma samples from the TARGET dataset, a log-rank test was conducted to compare the survival differences between the two or more groups in the KM survival analysis. Meanwhile, timeROC analysis was conducted to evaluate the accuracy of the prediction model. The least absolute shrinkage and selection operator (LASSO) regression algorithm was used for feature selection, and tenfold cross validation was performed. The above analysis was conducted using R software glmnet package. For the Kaplan–Meier curves, *p*-values and hazard ratios (HR) with a 95% confidence interval (CI) were obtained through log-rank test and univariate Cox regression. All the above statistical analyses and R software packages were executed using the version 4.0.3 of R software (R Foundation for Statistical Computing, 2020) [20], with a *p*-value less than 0.05 considered significant.

### Cell culture

The human neuroblastoma SH-SY5Y cell line was used in this study, which was purchased from SUNNCELL (Wuhan, China) and cultured in DMEM medium (Gibco, USA) containing 10% fetal bovine serum (Gibco, USA) at 5% CO_2_ at 37℃. The passage was conducted upon reaching 90% cell confluence.

### Cell viability assay

Puerarin was purchased from MedChemExpress (America, HY-N0145). ShSy-5Y cells were inoculated in 96-well plates at a density of 5000 cells per well and cultured in complete medium for 24 h. Subsequently, puerarin was added to the medium to achieve the final puerarin concentrations of 0, 25, 50, 75, 100, 150 and 200 µmol/L, and the cells were continuously cultured for 24 h. Each well was added with 10 μL CCK-8 solution (Abbkine, China) and incubated at 37℃ for 2 h. The absorbance at 450 nm was measured using enzyme-labeled instrument.

### Wound healing test

SH-SY5Y cells were seeded in a complete medium at a density of 20,000 cells per well in a six-well plate and cultured until reaching 85% confluence [21]. A scratch was made to create a wound upon reaching this confluence. After gentle washing, the cells were treated with puerarin at concentrations of 0, 50, 100, and 150 µM in DMEM medium without FBS. The wound surface areas were measured for the next 24 h to evaluate cell migration ability.

### Transwell test

SH-SY5Y cells were seeded at a density of 50,000 cells/well in the upper chamber of a transwell plate using serum-free DMEM diluted with puerarin at concentrations of 0, 50, 100, and 150 µM [21]. The lower wells were filled with complete DMEM culture medium containing 20% FBS to facilitate their migration. After a 24-h incubation in a cell culture container, the cells were fixed with 4% paraformaldehyde for 10 min. Following fixation, the cells in the upper chamber were delicately removed using a cotton swab, leaving behind the migrated cells, which were subsequently stained with crystal violet and imaged under a microscope.

### Colony‑forming assay

SH-SY5Y cells were seeded at a density of 500 cells per well in a six-well plate and cultured in complete cell culture medium for six hours to facilitate recovery. Different concentrations of puerarin, including end densities of 0, 50, 100, and 150 µM were added to the medium. Following two weeks of culture, the cells were gently washed, and the medium was aspirated. Subsequently, the cells were then fixed with 4% paraformaldehyde for 10 min, stained with crystal violet for 15 min, washed again, and finally observed under a microscope.

### Parachute assay

Sh-SY5Y cells were inoculated in 24-well plates and pre-cultured in complete medium containing puerarin at concentrations of 0, 50, 100, and 150 µM for 24 h. On the following day, some of the pores in each concentration were selected as donor cells. Calcein-AM was absorbed in complete culture medium containing 10 μmol/L Calcein-AM (Solarbio, Beijing), and transformed into calcein with green (530 nm) fluorescence signal. The cells were then incubated in darkness for 30 min. The recipient cells were cultured in 24-well plates until fully confluent. Following that, 500 Calcein-labeled donor cells were added to each well, and incubated in the dark for 3 h. [22] Then, the media containing donor cells were sucked away and rinsed 3 times with PSB. Fresh media was added, and random photos were taken using a fluorescence microscope (ti2-u, Nikon, Japan).

### Detection of mitochondrial membrane potential

The JC-1 kit from Beyotime, China was employed for mitochondrial membrane potential detection. SH-SY5Y cells were seeded at 5 × 103 cells/well in a 96-well plate. After 24 h, the cells were stained using the kit and observed under a fluorescence microscope. In conditions of high mitochondrial membrane potential, JC-1 formed red fluorescent J-aggregates in the mitochondrial matrix, while under low mitochondrial membrane potential, JC-1 remained in the green fluorescent monomeric form.

### Immunofluorescence (IF) staining

Cells were seeded onto slides in 24-well plates, and fixed in 4% paraformaldehyde for 20 min. Subsequently, they were immersed in 0.5% Triton-X in PBS for 10 min to enhance cell membrane permeability. The slides were then blocked with goat serum drops for 1 h at 37° C. Primary antibodies were added and left to incubate overnight at 4 °C. The next day, the cells were washed three times with PBST before adding the corresponding secondary antibodies. The slides were then incubated for 1 h at 37 °C. Subsequently, DAPI was used to label the nuclei. The cell slides were taken out of the 24-well plate and positioned on a glass slide pre-treated with an anti-fluorescence quencher. Finally, they were photographed under an upright fluorescence microscope for observation.

### qRT‑PCR

Cells at different intervention concentrations were homogenized and extracted using the TRIzol™ reagent (Ambion, 155,596–026) as per manufacturer's instructions. Reverse transcription was performed using PrimeScript RT Master Mix (Vazyme, China). Then, qRT-PCR was carried out using AceQ qPCR SYBR Green Master Mix (Vazyme, China), with GAPDH taken as the internal parameter. All primers were purchased from Sangon Biotech (Shanghai, China). The sequences of the primers were as follows: GADPH, 5′-GCACCGTCAAGGCTGAGAAC-3′ (sense) and 5′-TGGTGAAGACGCCAGTGGA-3′(antisense); BIRC5 forward, 5′- ATTCGTCCGGTTGCGCTTTCC -3′ (sense) and 5′- CACGGCGCACTTTCTTCGCAG-3′(antisense); TIMP2 forward, 5′-GCACATCACCCTCTGTGACT-3′ (sense) and 5′-CTGGTGCCCGTTGATGTTCT-3′(antisense); CASP9 forward, 5′- GAGGTGAAGAACGACCTGACTG-3′ (sense) and 5′- CTCAATGGACACGGAGCATC-3′(antisense); CX43 forward, 5′- TCTGCCTTTCGCTGTAACACT-3′ (sense); and 5′- GGGCACAGACACGAATATGAT-3′(antisense).

### Western blot

Protein samples were separated by sodium dodecyl sulfate–polyacrylamide gel electrophoresis and transferred to polyvinylidene difluoride (PVDF) membrane (Millipore, USA). The membrane was blocked with 5% nonfat dry milk for 2 h at room temperature and then incubated at 4 °C overnight with a primary antibody. Blots were washed three times in TBST for 10 min and incubated with the secondary antibodies at room temperature for 1 h. Protein detection was conducted using a Millipore ECL reagent with visualization achieved through the Tanon System, following three rounds of TBST washing. Quantification of data was achieved through GAPDH expression, and the analysis was performed using ImageJ software version 5.2.1. The antibodies used in this study are shown in Table 1.

**Table 1:** The antibodies and dilution ratios used for experiments

Antibody	Company	Batch number
JAML	abcam	ab183714
Cx43	abcam	ab312836
ROCK1	abcam	ab134181
RhoA	Proteintech	10,749–1-AP
MLCK	abcam	ab232949
p-CX43	Bioss	bs-3098R
GAPDH	Huabio	ET1601-4

### Statistical analysis

SPSS 27.0 was used for data analysis. Quantitative data were expressed as the mean ± SD of at least three independent experiments. Besides, one-way analysis of variance was conducted to compare the means. *P* < 0.05 was considered statistically significant.

## Results

### Potential target acquisition in neuroblastoma

**Fig. 1:**
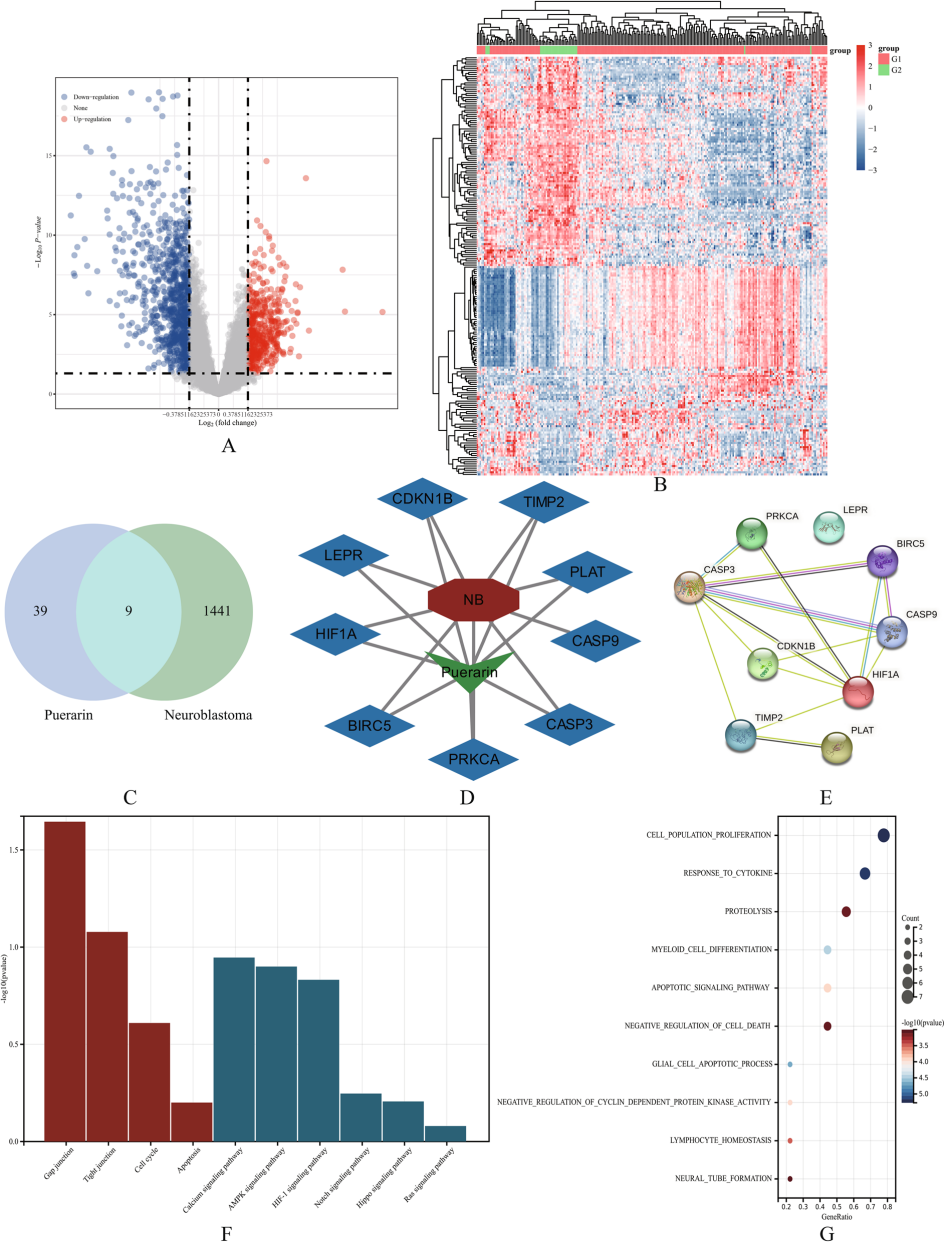
**A **A volcano plot drawn based on Fold change and corrected *p*-values. **B **Differential gene expression heatmap. **C **Venn diagram of the effective targets of Puerarin and potential therapeutic targets of neuroblastoma. **D** Drug-disease related targets containing 9 genes. **E** Protein–protein interaction (PPI) network of the common targets of puerarin and neuroblastoma. **F**, **G** KEGG and GO analysis of drug-disease related targets

RNA sequencing data and corresponding clinical information of 213 COG high-risk group and 31 COG low-risk group patients were obtained from the TARGET database, and the differentially expressed genes between the two groups were analyzed. A total of 1,451 differentially expressed genes were ultimately obtained as potential targets for neuroblastoma. As shown in Fig. 1A, the red dots in the diagram represent significantly upregulated genes, the blue dots represent significantly downregulated genes, and the gray dots represent insignificant genes. Differential gene expression heatmap is shown in Fig. 1B, where different colors represent expression trends in different tissues. Due to the large number of differential genes, the top 50 upregulated and downregulated genes with the largest differential changes are separately presented here.

### Drug-disease related targets acquisition

Herein, 48 potential targets for puerarin were identified from the TCMSP database. Through the Venny R package, 9 key-target genes were extracted and illustrated in a Venn diagram (Fig. 1C). Additionally, the outcomes were visualized as a complex drug-disease target network (Fig. 1D).

### Construction of the PPI network

To further understand the relationship between various drug-disease related targets, a STRING database and the restricted species Homo sapiens were employed to obtain a PPI network (Fig. 1E).

### GO and KEGG pathway enrichment analyses

In this study, 9 key-target genes were analyzed using the Bioconductor library in R for GO and KEGG pathway enrichment analyses. The top 10 results were obtained and visualized as bar charts and bubble charts. As shown in Fig. 1F, KEGG analysis obtained Gap junction, Tight junction, cell cycle, etc., while GO analysis obtained cell population proliferation, response to cytokine, proteolysis, etc., as shown in Fig. 1G.

### Correlation test between genes and survival

**Fig. 2:**
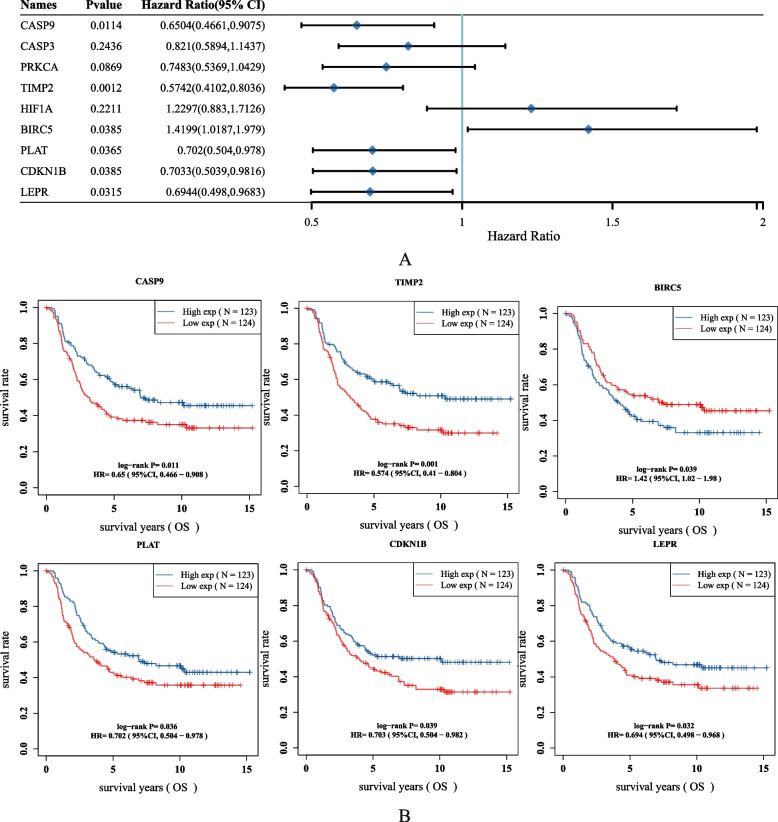
**A** Distribution of KM survival curves of 6 drug-disease related genes related to survival in the TARGET dataset. **B **Association of 9 drug-disease related genes with neuroblastoma survival

The correlation between 9 key target genes and survival was tested, and the results showed that 6 genes were significantly associated with neuroblastoma survival (Fig. 2B). As shown in Fig. 2A, BIRC5 was negatively correlated with survival, while CASP9, TIMP2, PLAT, CDKN1B and LEPR demonstrated a positive correlation with survival.

### Construction of a prognostic model

**Fig. 3:**
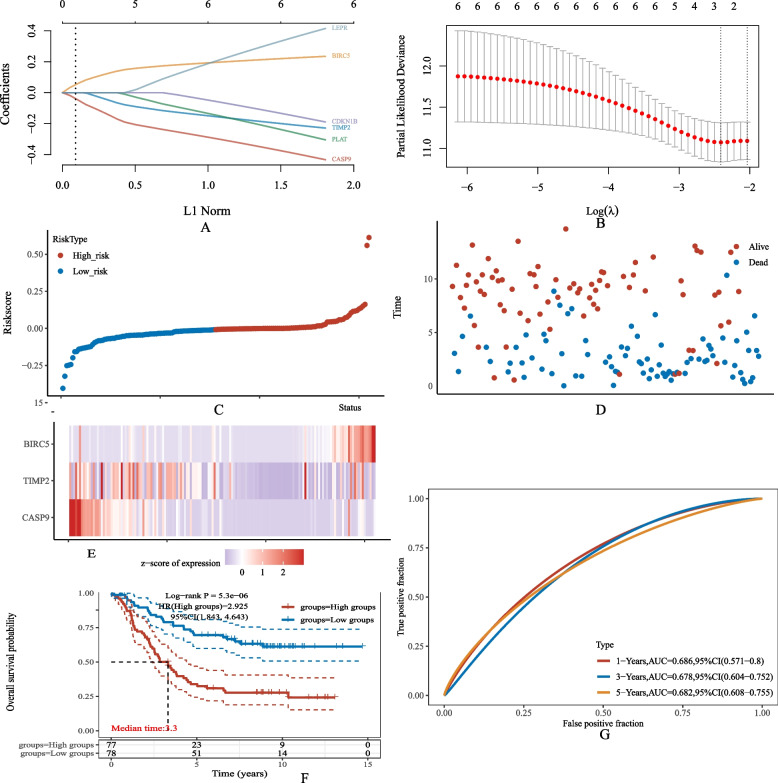
**A**, **B** The coefficients of selected features are shown by lambda parameter. The abscissa represents the value of lambda, and the ordinate represents the coefficients of the independent variable. **D **Scatterplot represents the Riskscore from low to high. **C **Scatterplot distribution represents the Riskscore of different samples correspond to the survival time and survival status. **E** Heatmap of the gene expression from the signature. **F **KM survival curves of the prognostic model in the Target dataset. **G** ROC curves and AUC values of prognostic model at 1, 3 and 5 years

LASSO COX regression analysis of these 6 genes demonstrated stable and robust performance (Fig. 3A, B). In order to obtain the optimal results, multivariate COX regression analysis was performed on 6 key-target genes, and 3 genes associated with neuroblastoma were identified, i.e., BIRC5, TIMP2 and CASP9. A risk score was calculated for each patient based on mRNA expression levels and risk coefficients for 3 key target genes. According to the median risk score, patients with higher than the median score were divided into the high-risk group and the low-risk group to predict the prognosis of neuroblastoma patients (Fig. 3C). As shown in Fig. 3D, the mortality rate of the high-risk group was observed to be higher than that of the low-risk group, while the survival rate was lower than that of the low-risk group. Subsequently, the expression heat maps of 3 key target genes in high-risk and low-risk groups were constructed. As shown in Fig. 3E, the expression of BIRC5 in the high-risk group was higher than that in the low-risk group, confirming these genes as high-risk ones. Meanwhile, the expression of TIMP2 and CASP9 in the high-risk group was lower than that in the low-risk group, demonstrating these genes as low-risk ones. Kaplan–Meier analysis showed that the survival rate of the high-risk group was lower than that of the low-risk group at any time (Fig. 3F). In order to further evaluate the predictive effect of the prognostic model, the ROC curve changing over time was used for analysis. As shown in Fig. 3G, the 1-year AUC was 0.686, the 2-year AUC was 0.678, and the 3-year AUC was 0.682, indicating the good predictive ability of the model.

### Puerarin inhibiting the activity and proliferation of SH-SY5Y cells

**Fig. 4:**
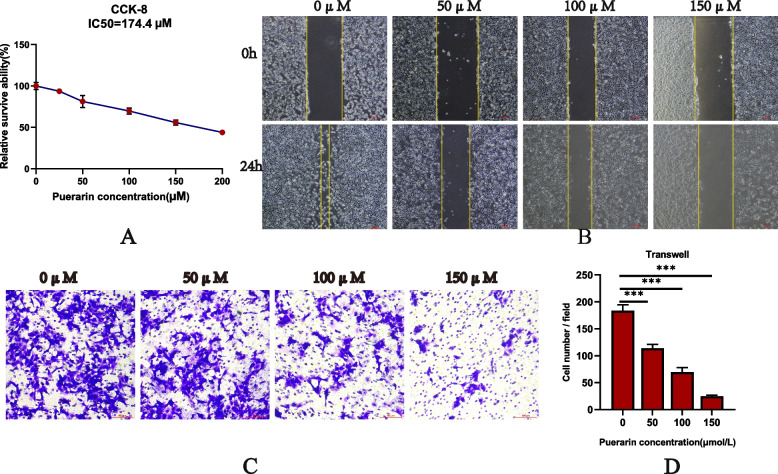
**A **With the increase of puerarin concentration, SH-SY5Y cells activity decreased gradually and reached IC50 at 174.4 μM. **B** Puerarin inhibited SH-SY5Y cell migration in wound healing tests. **C** and **D** Transwell experiment showed that puerarin could inhibit the invasion ability of SH-SY5Y cells, ***P < 0.001

**Fig. 5:**
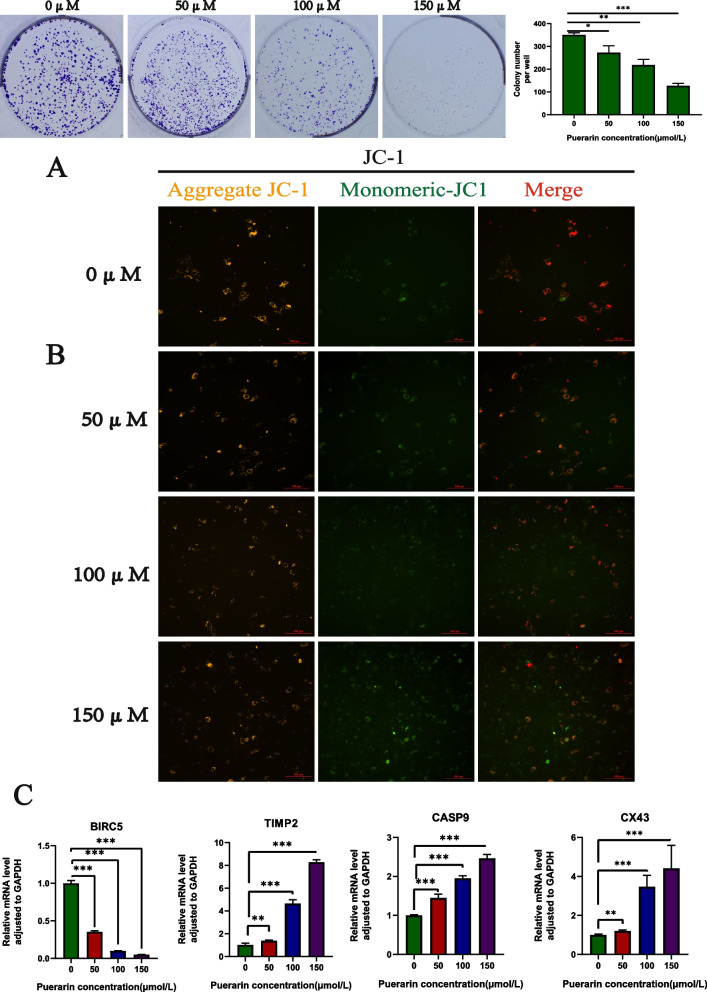
**A** Colony formation experiment showed that puerarin could inhibit the proliferation of SH-SY5Y cells, and the inhibitory ability became stronger with the increase of concentration within 150 µmol/L* *P* < 0.05, ** *P* < 0.01, and ***P < 0.001. **B** As the dose of puerarin increased, the fluorescence intensity of aggregated JC-1 gradually diminished, accompanied by a reduction in the number of cells labeled red. **C** Puerarin could significantly down-regulate the expression of BIRC5 in SH-SY5Y cells while significantly up-regulating the levels of CX43, TIMP2 and CASP9

Cell viability was hereby assessed using the CCK8 method, and the results showed that with the increase of concentration, the activity of SH-SY5Y cells decreased gradually and reached IC50 at 174.4 μM (Fig. 4A). Therefore, in order to prevent cell death from impacting the outcomes of other experiments, 150 μM was chosen as the highest experimental concentration to ensure cell viability. Besides, colony formation experiments were performed to evaluate the effect of puerarin on the proliferation of SH-SY5Y cells, and colony formation experiments were carried out to assess the effect of puerarin on the proliferation of SH-SY5Y cells. As expected, compared with the 0 µM puerarin group, the cell colony numbers of 50, 100, and 150 µM groups were significantly reduced (*P* < 0.05) (Fig. 5A), thereby proving the efficacy of puerarin in inhibiting the proliferation of SH-SY5Y cells.

### Puerarin inhibiting the migration and invasion ability of SH-SY5Y cells

The wound healing experiment can reflect the migration ability of SH-SY5Y cells under different concentrations of stimulation. The results showed that the migration distance of SH-SY5Y cells decreased significantly with the increase of puerarin concentration (*P* < 0.05) (Fig. 4 B). Similarly, Transwell results showed that compared with the 0µMpuerarin groups, the cells on the lower surface of the 50,100 and 150µMpuerarin groups were significantly reduced, the higher the concentration of puerarin, the less the number of cells on the lower surface (*P* < 0.001). (Fig. 4C, D) This indicated that puerarin could inhibit the migration ability of SH-SY5Y cells.

### Puerarin increased mitochondrial damage in SH-SY5Y cells

Mitochondria serve as vital organelles for biological energy production and biosynthesis, playing a crucial role in tumor cell metabolism, growth, and survival. To assess puerarin's impact on mitochondrial function, SH-SY5Y cells were hereby stained with JC-1 at different intervention concentrations. As shown in Fig. 5B, with the increase of dose, the fluorescence intensity of aggregate JC-1 decreased gradually, and the number of cells labeled red decreased as well. The results showed that puerarin caused mitochondrial damage to SH-SY5Y cells.

### Puerarin downregulating the expression of BIRC5 while upregulating that of TIMP2 and CASP9 in SH-SY5Y cells

As shown in Fig. 5C, puerarin could significantly down-regulate the expression of BIRC5 while significantly up-regulating the expression of TIMP2 and CASP9 in SH-SY5Y cells. The trend was increasingly obvious with the increase of puerarin concentration within 150 µmol/L.

### Puerarin enhancing gap junction intercellular communication of SH-SY5Y cells

**Fig. 6:**
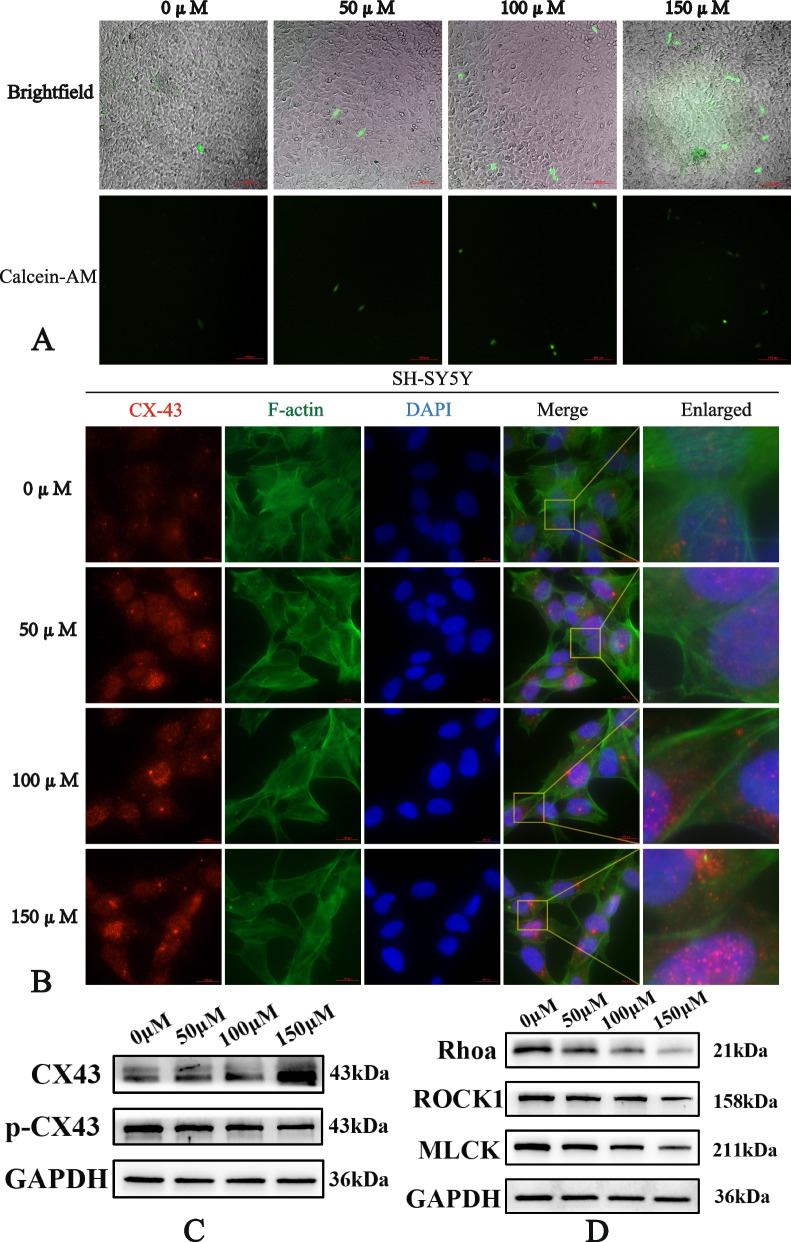
**A** Parachute assay showed that the number of receptor cells receiving the calcein signal increased with the increase of puerarin concentration. **B** Immunofluorescence showed that with the increase of puerarine concentration, the cytoskeleton became blurred and disordered, the pseudopod structure around the cells decreased, the fluorescence intensity of CX-43 increased, and abundant expression of CX-43 was observed at the cell junction. **C** Puerarin could enhance the expression of CX-43 in SH-SY5Y cells and inhibit its phosphorylation. The image is cropped, full-length blots are presented in Supplementary Figure WB. **D** Puerarin inhibited the expression of Rhoa, ROCK1 and MLCK in the RhoA-ROCK1 signaling pathway. The image is cropped, full-length blots are presented in Supplementary Figure WB

Since gap junction was identified as significant in the KEGG analysis, the impact of puerarin on this pathway was further investigated. Gap junctional intercellular communication (GJIC) serves as one of the most common forms of communication between cells. GJIC is closely related to tumor, with most tumor cells exhibiting decreased or impaired GJIC capability, reduced connexin expression, or abnormal cellular localization. The shedding, invasion and metastasis of tumor cells are all linked to the abnormality of GJIC, while restoration of GJIC can impede tumor progression. As shown in Fig. 6A, the number of receptor cells receiving calcein signals increased with the increase of puerarin concentration. This showed that GJIC of SH-SY5Y cells was progressively strengthened with increasing puerarin concentration.

### Puerarin weakening the cytoskeleton of SH-SY5Y cells

Cytoskeletal proteins are involved in the migration and invasion of tumor cells. In order to further investigate the mechanism of inhibiting the migration and invasion of SH-SY5Y cells by pularin, immunofluorescence staining was hereby performed on F-actin cytoskeleton protein and Connexin 43 gap junction protein to identify the changes of cytoskeleton and GJIC. As shown in Fig. 6B, the cytoskeleton of the blank group was clear and arranged in an orderly way, and the pseudopod structure was extended around the cells to facilitate cell movement. The fluorescence expression of CX-43 appeared weak and primarily localized within the cells, with limited distribution at cell junctions initially. However, as the concentration of puerarin increased, the cytoskeleton became blurred and disordered, the pseudopod structure around the cells diminished, and the fluorescence intensity of CX-43 intensified. Additionally, abundant expression of CX-43 was observed at the cell junctions.

### Western blot

Western blot analysis was conducted to detect changes in the expression levels of key GJIC proteins. The results showed that puerarin could increase the expression of CX-43 in SH-SY5Y cells and inhibit its phosphorylation. Within 150 µmol/L, the trend became more obvious with the increase of puerarin concentration (Fig. 6C).

The Rhoa/ROCK1 signaling pathway is believed to regulate myoglobin and thereby regulate cytoskeletal remodeling and cell migration. In this study, the Western blot results showed that Rhoa, ROCK1 and MLCK gradually decreased with the increase of puerarin concentration. This suggested that puerarin might inhibit the motility of SH-SY5Y cells by inhibiting Rhoa/ROCK1 signaling pathway (Fig. 6D).

## Discussion

Neuroblastoma, among the most prevalent solid tumors in children, is marked by a concealed onset site, rapid tumor proliferation, and susceptibility to metastasis. Many children with high-risk neuroblastoma exhibit either no response to initial treatment or a high likelihood of relapse [8]. Therefore, it is particularly important to explore new drug small molecules for the treatment of neuroblastoma and establish a new differential gene prognosis model. As society and medicine evolve, traditional Chinese medicine has increasingly showcased its therapeutic benefits in disease treatment. Puerarin, as a small molecule drug extracted from Puerarin, has been shown to induce apoptosis of tumor cells, interfere with mitochondrial regulation, and damage mitochondria in a variety of tumors [23, 24]. However, its effects in neuroblastoma still need to be further explored and verified. Therefore, network pharmacology and bioinformatics methods, combined with in vitro experiments, were hereby utilized to analyze the possibility of puerarin application in neuroblastoma, and a prediction model was established.

The main puerarin targets were identified through the drug-ingredient-target interaction network. As indicated by KEGG analysis, the effect of puerarin on neuroblastoma might be realized by influencing Gap junction, Tight junction, cell cycle, etc. The GO analysis results suggested that, the effect of puerarin on neuroblastoma might be related to cell population proliferation, response to cytokine, proteolysis, etc. Therefore, it was hereby speculated that puerarin's impact on neuroblastoma might involve the inhibition of cell migration and movement through gap junctions, thereby suppressing neuroblastoma cell proliferation. Besides, multivariate COX regression analysis was carried out to obtain three genes related to the prognosis of neuroblastoma, namely BIRC5, TIMP2 and CASP9. Among them, BRIC5 is a high-risk gene, while TIMP2 and CASP9 are low-risk genes. Prior research has demonstrated that BIRC5 is notably up-regulated in neuroblastoma, and the down-regulation of BIRC5 induces apoptosis in neuroblastoma through mitotic catastrophe [25]. TIMP2 belongs to the family of Tissue inhibitors of metalloproteinases, and there are conflicting reports on its role in cancer, which is closely related to the invasion and migration of tumor cells [26]. Meanwhile, CASP9 is one of the key proteins in cell apoptosis, and its lack of expression can cause the development of various tumors and poor prognosis [27, 28]. Herein, the research results showed that puerarin could significantly down-regulate the mRNA expression levels of BIRC5 while significantly up-regulating the mRNA expression levels of TIMP2 and CASP9 in SH-SY5Y cells, which was consistent with the prediction model proposed.

To further verify the conjecture, wound healing experiment and transwell experiment were conducted to confirm the effects of puerarin on the migration and invasion of SH-SY5Y cells. The results showed that the migration distance of SH-SY5Y cells decreased significantly with the increase of puerarin concentration in wound healing experiment. In the transwell experiment, puerarin also inhibited the migration of SH-SY5Y cells. At the same time, CCK-8 and colony formation experiments were also carried out to verify whether puerarin could affect the cell proliferation of neuroblastoma. The findings revealed that as the concentration of puerarin increased, the cell viability of SH-SY5Y cells gradually declined, accompanied by a significant reduction in the number of colony formations. These results indicated that puerarin could inhibit the proliferation and migration of SH-SY5Y cells.

Based on the KEGG results, it was further speculated that the effect of puerarin on the migration ability of neuroblastoma might be mediated by the regulation of Gap junction. To further investigate the mechanism by which puerarin inhibited neuroblastoma migration, Parachute Assay and immunofluorescence staining were performed. The results showed that the number of positive cells in Parachute Assay increased with the increase of puerarin concentration, demonstrating the gradual recovery of GJIC with the increase of puerarin concentration in SH-SY5Y cells. According to the morphology and distribution of cytoskeleton and CX-43, immunofluorescence staining showed that the cytoskeleton could inhibit the cell motility of SH-SY5Y cells, while the intercellular expression and distribution of CX-43 could be up-regulated. This might enhance intercellular connections and mitigate neuroblastoma metastasis. Additionally, the effects of puerarin on mRNA expression of CX43 were detected by q-PCR, and it was found that with the increase of puerarin concentration, the expression level of CX43 mRNA in SH-SY5Y cells increased. These results indicated that puerarin could promote the mRNA expression of CX43. This might be attributed to the regulatory function of RhoA/ROCK signaling pathway on CX43 expression [29]. Furthermore, Western blots results of RhoA, ROCK1 and MLCK confirmed that puerarin could inhibit the activity of RhoA/ROCK signaling pathway in SH-SY5Y cells. These results indicated that puerarine could increase the expression of CX43 by regulating RhoA/ROCK signaling pathway, and thus enhance the GJIC of SH-SY5Y cells.

Previous reports have suggested that puerarin induces mitochondrial damage, contributing to its anti-tumor effects. In the present study, as puerarin dosage increased, JC-1 fluorescence intensity declined, accompanied by a reduction in red-labeled cells, indicating mitochondrial damage in SH-SY5Y cells.

## Conclusions

In summary, based on network pharmacology and bioinformatics analysis, combined with in vitro experimental verification, puerarin was hereby observed to enhance GJIC in neuroblastoma, destroy cytoskeleton and thus inhibit cell invasion and migration, cause mitochondrial damage of tumor cells, and inhibit cell proliferation. Overall, puerarin, as a natural medicinal compound, does hold potential as a novel therapy for neuroblastoma. However, its mechanism of action warrants further investigation and exploration.

### Supplementary Information

Supplementary Material 1.

Supplementary Material 2.

Supplementary Material 3.

Supplementary Material 4.

Supplementary Material 5.

Supplementary Material 6.

Supplementary Material 7.

Supplementary Material 8.

## Data Availability

The data for this study come from TARGET database (https://ocg.cancer.gov/programs/target) and TCMSP database (https://old.tcmsp-e.com/tcmsp.php). All the data in this paper support the results of this study.

## References

[1] London WB, Castleberry RP, Matthay KK (2005). Evidence for an age cutoff greater than 365 days for neuroblastoma risk group stratification in the Children's Oncology Group. J Clin Oncol.

[2] Gatta G, Botta L, Rossi S (2014). Childhood cancer survival in Europe 1999–2007: results of EUROCARE-5–a population-based study [published correction appears in Lancet Oncol. 2014 Feb;15(2): e52]. Lancet Oncol.

[3] Tolbert VP, Matthay KK (2018). Neuroblastoma: clinical and biological approach to risk stratification and treatment. Cell Tissue Res.

[4] Strother DR, London WB, Schmidt ML (2012). Outcome after surgery alone or with restricted use of chemotherapy for patients with low-risk neuroblastoma: results of Children's Oncology Group study P9641. J Clin Oncol.

[5] Kohler JA, Rubie H, Castel V (2013). Treatment of children over the age of one year with unresectable localised neuroblastoma without MYCN amplification: results of the SIOPEN study. Eur J Cancer.

[6] Matthay KK, Villablanca JG, Seeger RC (1999). Treatment of high-risk neuroblastoma with intensive chemotherapy, radiotherapy, autologous bone marrow transplantation, and 13-cis-retinoic acid. Children's Cancer Group. N Engl J Med.

[7] Pinto NR, Applebaum MA, Volchenboum SL (2015). Advances in risk classification and treatment strategies for neuroblastoma. J Clin Oncol.

[8] London WB, Castel V, Monclair T (2011). Clinical and biologic features predictive of survival after relapse of neuroblastoma: a report from the International Neuroblastoma Risk Group project. J Clin Oncol.

[9] Liu X, Huang R, Wan J (2023). Puerarin: a potential natural neuroprotective agent for neurological disorders. Biomed Pharmacother.

[10] Yu L, Gao F, Yang L, Xu L, Wang Z, Ye H (2012). Biotransformation of puerarin into puerarin-6″-O-phosphate by Bacillus cereus. J Ind Microbiol Biotechnol.

[11] Qin W, Guo J, Gou W (2022). Molecular mechanisms of isoflavone puerarin against cardiovascular diseases: what we know and where we go. Chin Herb Med.

[12] Gao M, Zhang Z, Lai K (2022). Puerarin: a protective drug against ischemia-reperfusion injury. Front Pharmacol.

[13] Cai D, Zhao Y, Yu F (2022). Puerarin ameliorates acute lung injury by modulating NLRP3 inflammasome-induced pyroptosis. Cell Death Discov..

[14] Semenov AL, Tyndyk ML, Von JD (2023). Effects of isoflavone-rich nades extract of pueraria lobata roots and astaxanthin-rich phaffia rhodozyma extract on prostate carcinogenesis in rats. Plants (Basel).

[15] Lang J, Guo Z, Xing S (2022). Inhibitory role of puerarin on the A549 lung cancer cell line. Transl Cancer Res.

[16] Cai Y, Gao Q, Meng JH, Chen L (2023). Puerarin suppresses glycolysis and increases cisplatin chemosensitivity in oral squamous cell carcinoma via FBXW7/mTOR signaling. Nutr Cancer.

[17] Wang Q, Shen ZN, Zhang SJ, Sun Y, Zheng FJ, Li YH (2022). Protective effects and mechanism of puerarin targeting PI3K/Akt signal pathway on neurological diseases. Front Pharmacol..

[18] Zhu X, Wang K, Zhang K, Lin X, Zhu L, Zhou F (2016). Puerarin Protects Human Neuroblastoma SH-SY5Y Cells against Glutamate-Induced Oxidative Stress and Mitochondrial Dysfunction. J Biochem Mol Toxicol.

[19] Zhao J, Wang J, Liu J, Li S, Liu P, Zhang X (2022). Effect and mechanisms of kaempferol against endometriosis based on network pharmacology and in vitro experiments. BMC Complement Med Ther..

[20] Lin Z, Sui X, Jiao W, Chen C, Zhang X, Zhao J (2022). Mechanism investigation and experiment validation of capsaicin on uterine corpus endometrial carcinoma. Front Pharmacol..

[21] Wu S, Wu Z, Xu H (2022). miR-34a-5p inhibits the malignant progression of KSHV-infected SH-SY5Y cells by targeting c-fos. PeerJ..

[22] Gingrich J, Pu Y, Veiga-Lopez A (2021). A modified parachute assay for assessment of gap junction intercellular communication in placental trophoblast cells. Toxicol Mech Methods.

[23] Li J, Xiong C, Xu P, Luo Q, Zhang R (2021). Puerarin induces apoptosis in prostate cancer cells via inactivation of the Keap1/Nrf2/ARE signaling pathway. Bioengineered.

[24] Liu X, Zhao W, Wang W, Lin S, Yang L (2017). Puerarin suppresses LPS-induced breast cancer cell migration, invasion and adhesion by blockage NF-κB and Erk pathway. Biomed Pharmacother.

[25] Lamers F, van der Ploeg I, Schild L (2011). Knockdown of survivin (BIRC5) causes apoptosis in neuroblastoma via mitotic catastrophe. Endocr Relat Cancer.

[26] Kai AK, Chan LK, Lo RC (2016). Down-regulation of TIMP2 by HIF-1α/miR-210/HIF-3α regulatory feedback circuit enhances cancer metastasis in hepatocellular carcinoma. Hepatology.

[27] Ronellenfitsch MW, Oh JE, Satomi K (2018). CASP9 germline mutation in a family with multiple brain tumors. Brain Pathol.

[28] Zhang M, Wu K, Wang M, Bai F, Chen H (2022). CASP9 as a prognostic biomarker and promising drug target plays a pivotal role in inflammatory breast cancer. Int J Anal Chem..

[29] Gómez GI, Velarde V, Sáez JC (2019). Role of a RhoA/ROCK-dependent pathway on renal connexin43 regulation in the angiotensin II-induced renal damage. Int J Mol Sci..

