# Supplementary figures and images for: Effects and mechanisms of puerarin against neuroblastoma: insights from bioinformatics and in vitro experiments
Source: BMC Complement Med Ther. 2024 Jul 9;24:257. doi: 10.1186/s12906-024-04569-0 (PMC11234716; doi:10.1186/s12906-024-04569-0)

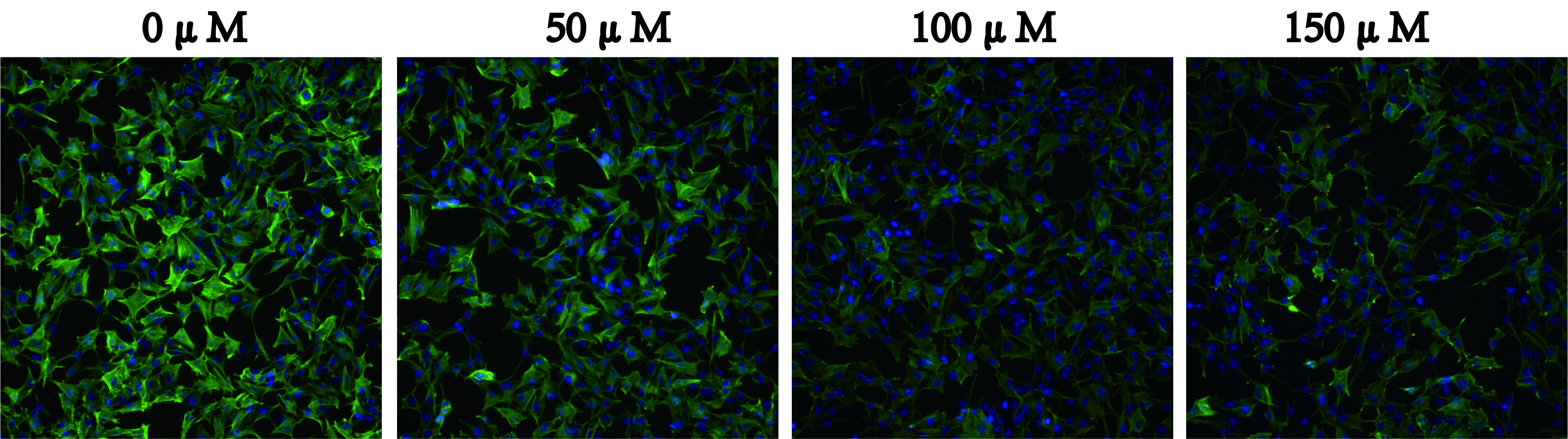

Supplement: Supplementary file 1 — Supplementary Material 1. [file 12906_2024_4569_MOESM1_ESM.tif]

**CX43**

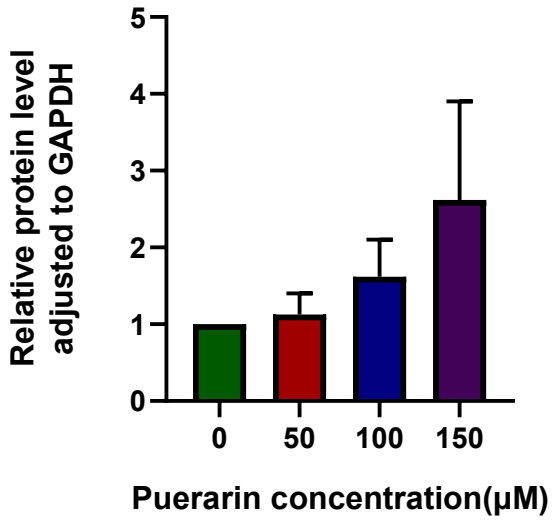

**p-CX43**

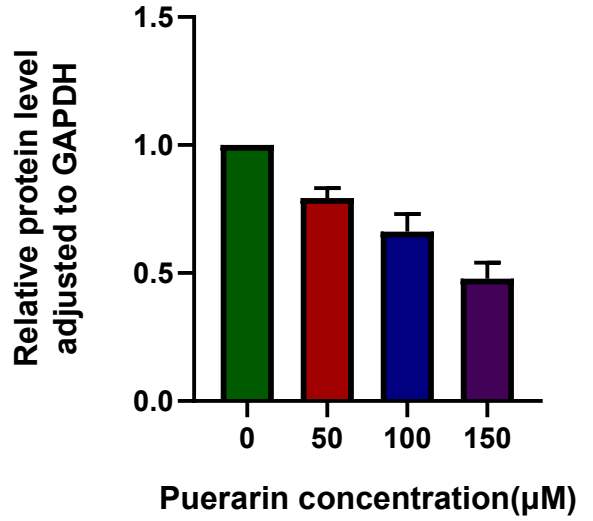

**RhoA**

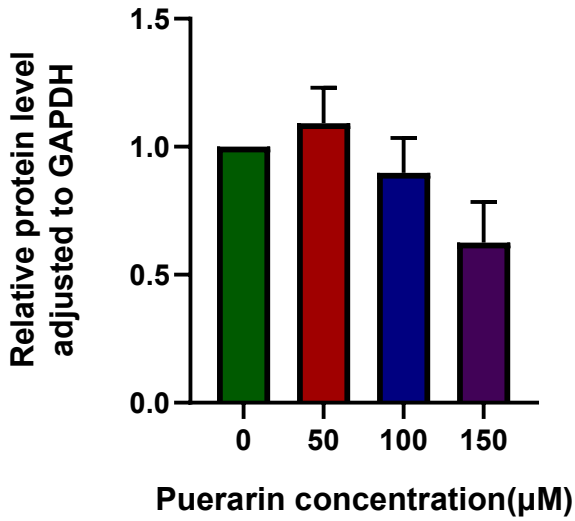

**RhoA**

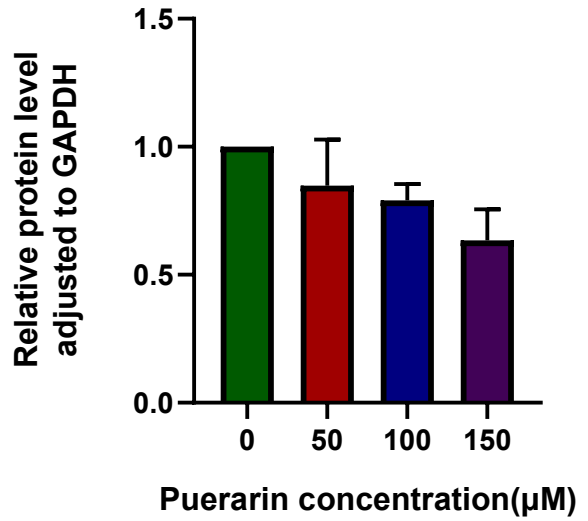

**MLCK**

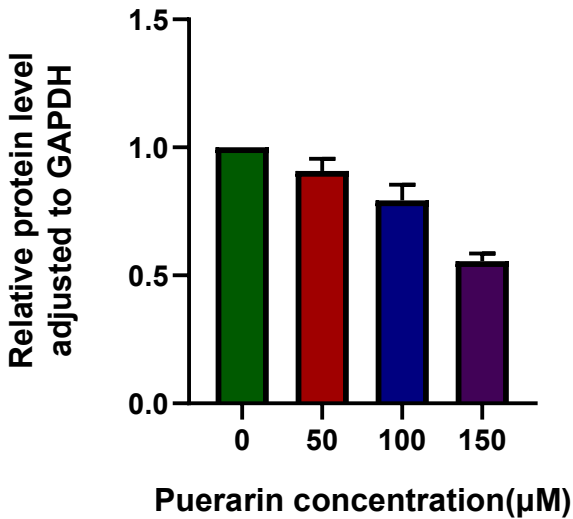

Supplement: Supplementary file 3 — Supplementary Material 3. [file 12906_2024_4569_MOESM3_ESM.pdf]

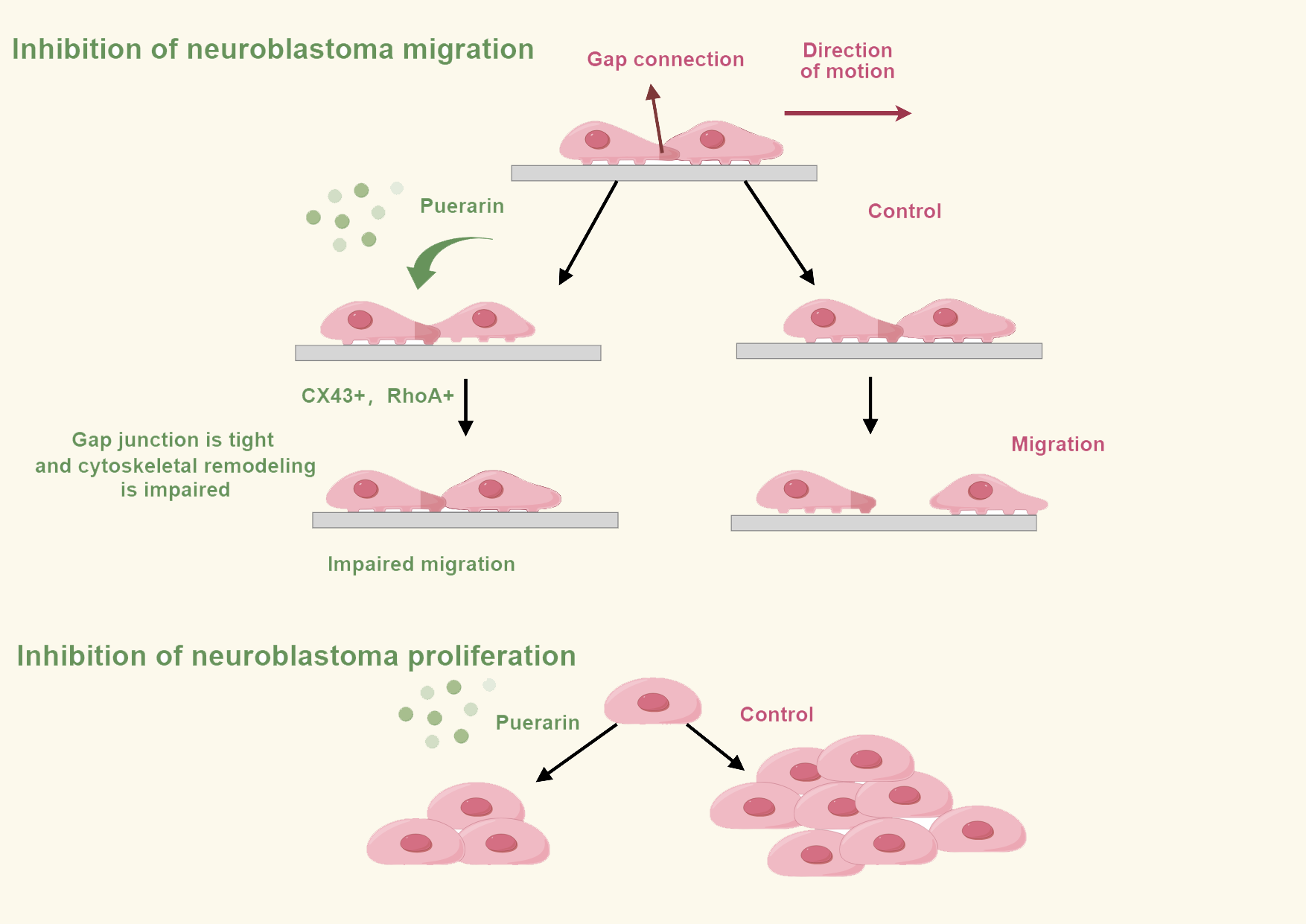

Supplement: Supplementary file 4 — Supplementary Material 4. [file 12906_2024_4569_MOESM4_ESM.png]

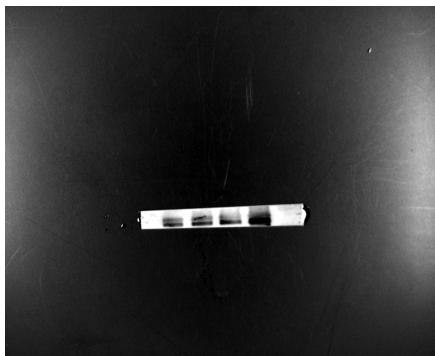

CX43

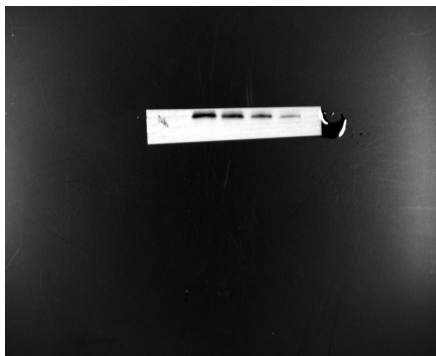

RhoA

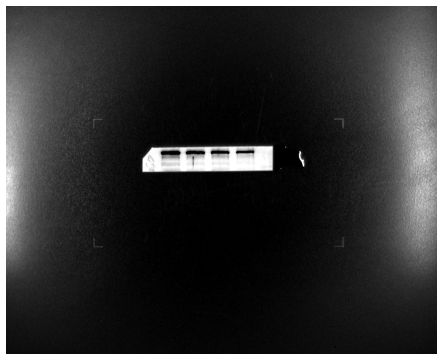

p-CX43

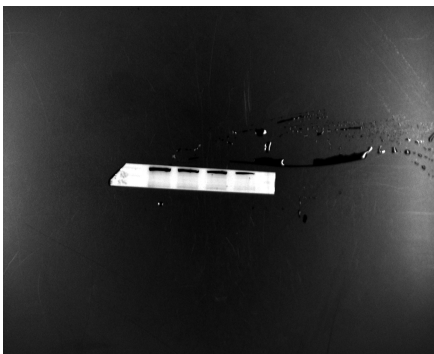

ROCK1

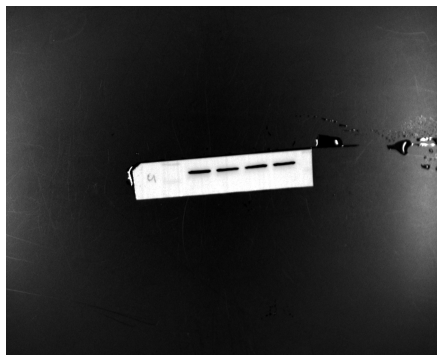

GAPDH

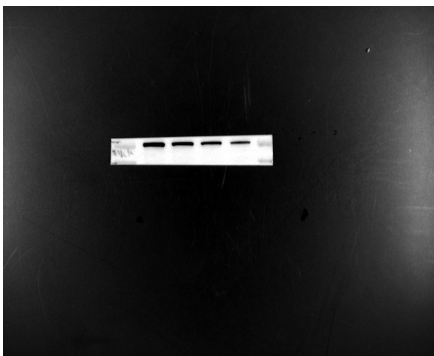

MLCK

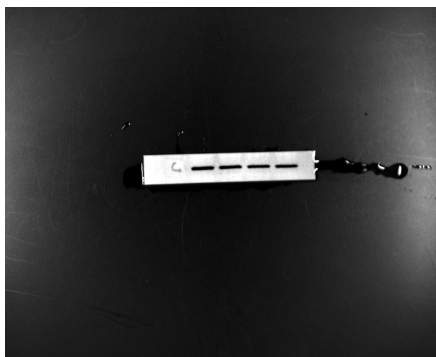

GAPDH

Supplement: Supplementary file 7 — Supplementary Material 7. [file 12906_2024_4569_MOESM7_ESM.pdf]

**0  $\mu\text{M}$**

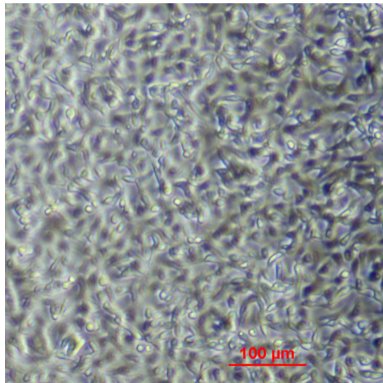

**50  $\mu\text{M}$**

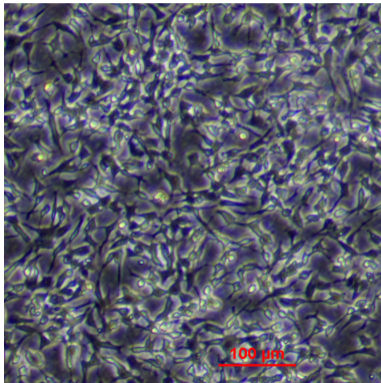

**100  $\mu\text{M}$**

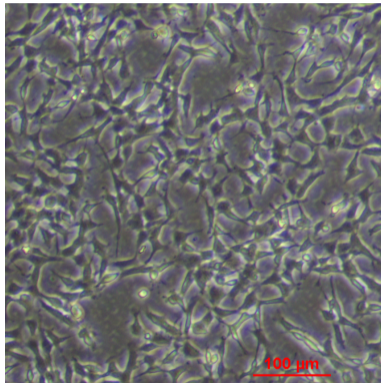

**150  $\mu\text{M}$**

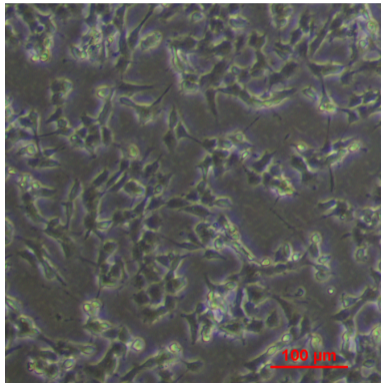

Supplement: Supplementary file 8 — Supplementary Material 8. [file 12906_2024_4569_MOESM8_ESM.pdf]
