# Supplementary material for: Effects and mechanisms of puerarin against neuroblastoma: insights from bioinformatics and in vitro experiments
Source: BMC Complement Med Ther. 2024 Jul 9;24:257. doi: 10.1186/s12906-024-04569-0 (PMC11234716; doi:10.1186/s12906-024-04569-0)

The original image of the content in the figure

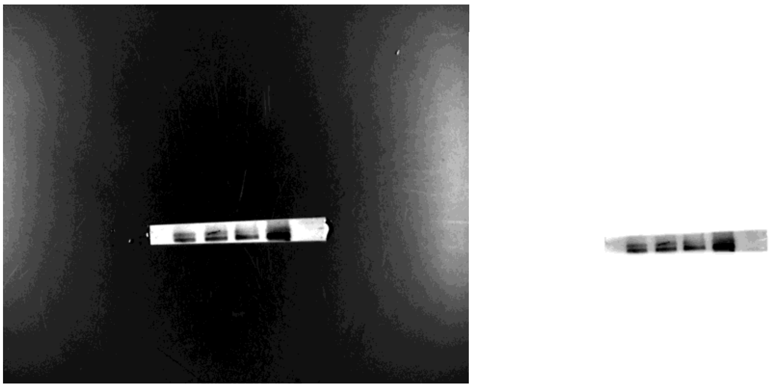

CX-43 (Clipping retention 55-40Kda)

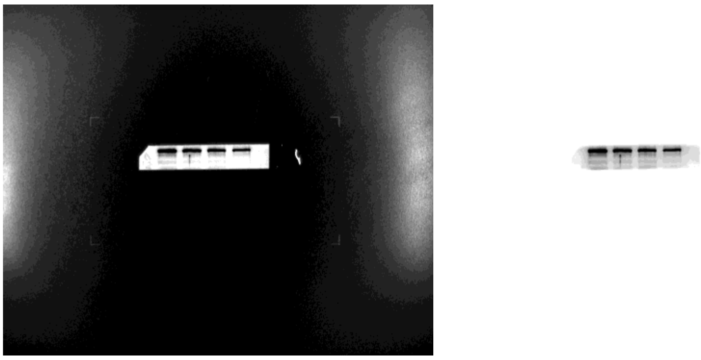

PCX-43 (Clipping retention 55-40Kda)

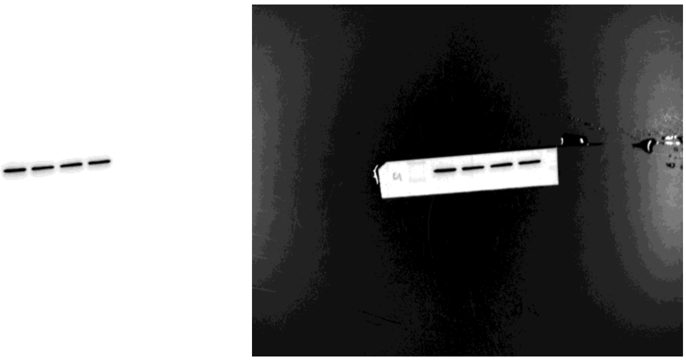

GAPDH (Clipping retention 40-35Kda)

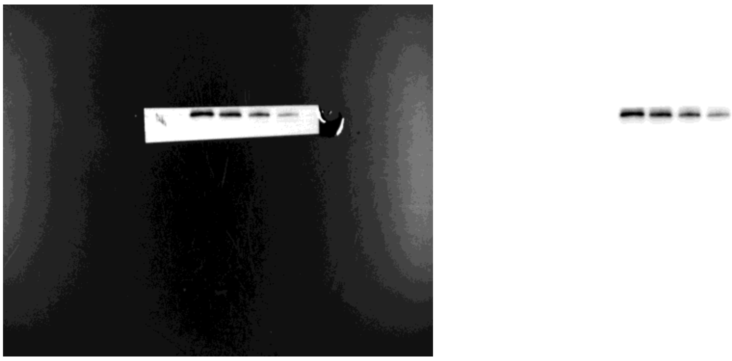

Rhoa (Clipping retention 25-15Kda)

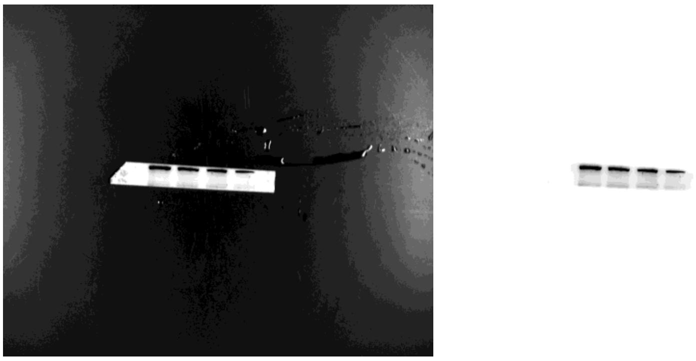

ROCK1 (Clipping retention 180-100Kda)

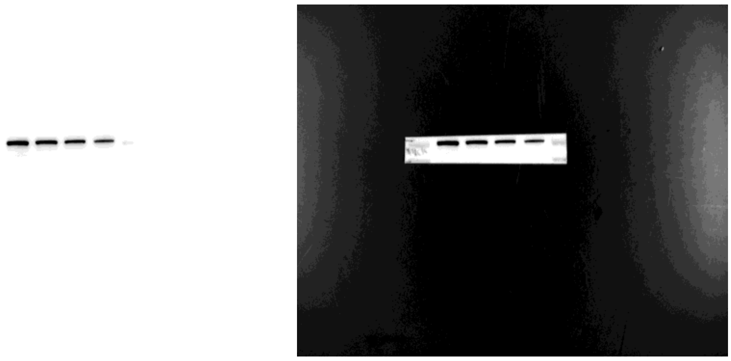

MLCK1 (Clipping retention 180-100Kda)

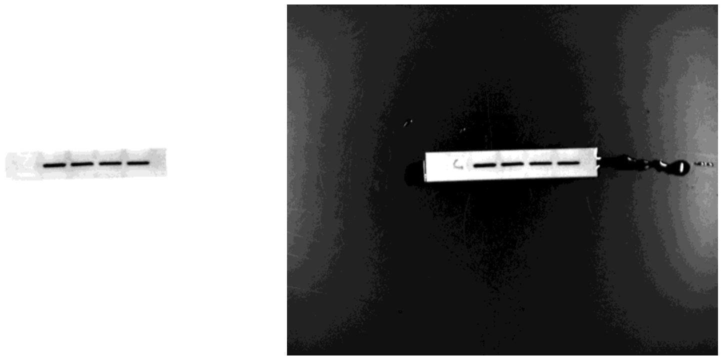

GAPDH (Clipping retention 40-35Kda)

Rest of the pictures

CX-43

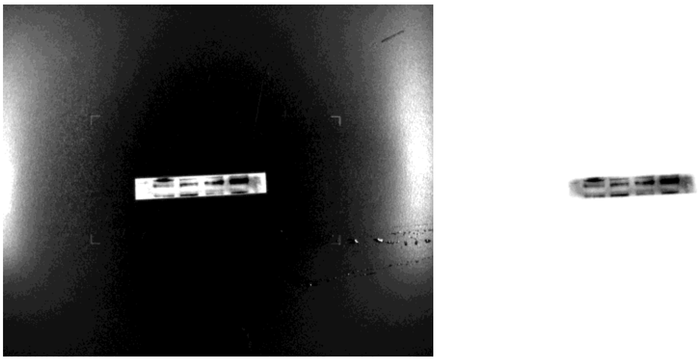

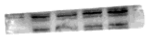
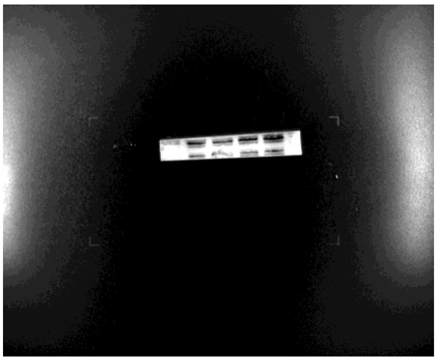

PCX-43

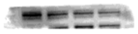
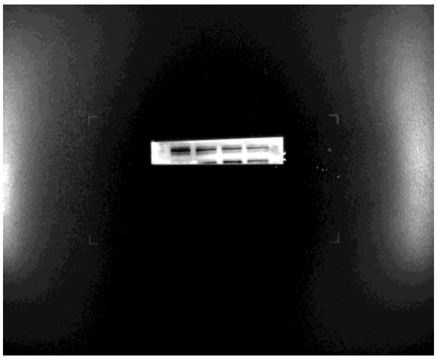

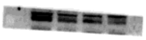
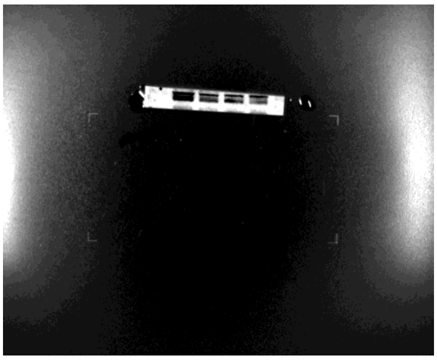

Rhoa

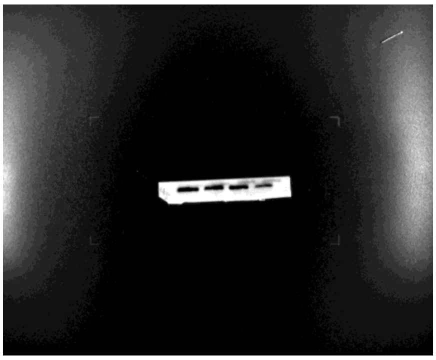
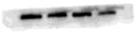

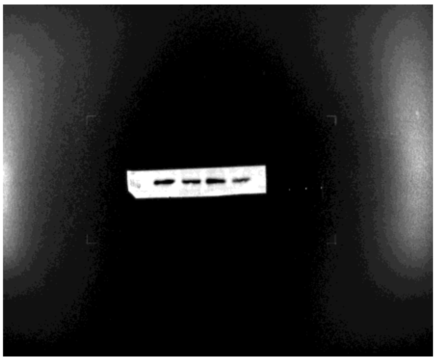
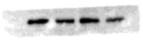

ROCK1

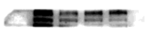
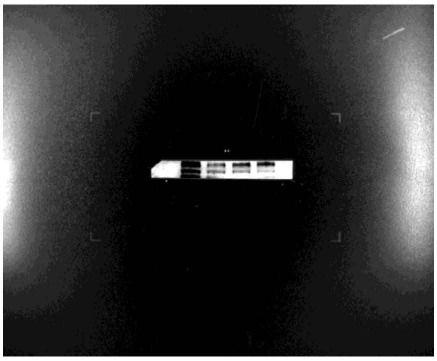

MLCK

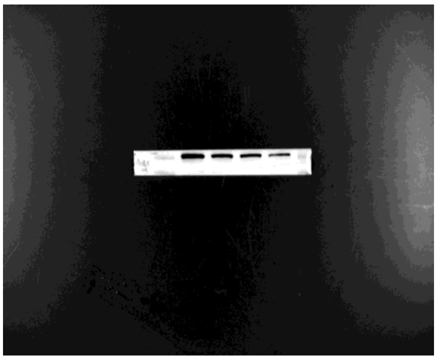

GAPDH

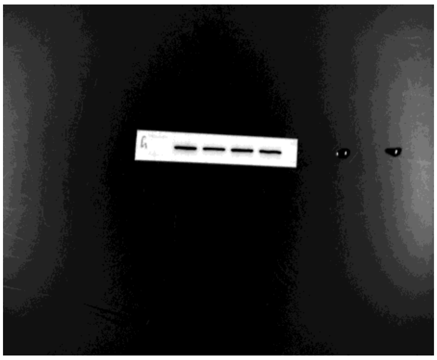

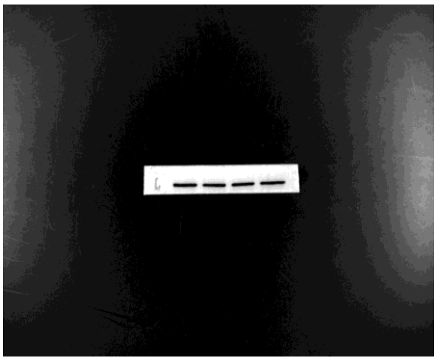

Supplement: Supplementary file 5 — Supplementary Material 5. [file 12906_2024_4569_MOESM5_ESM.pdf]
